# The Pathological Society

**Published:** 1897-02-20

**Authors:** 


### THE PATHOLOGICAL SOCIETY.

At the meeting held on February 16th an interesting lantern demonstration was given by Dr. T. C. Fox and Dr. F. R. Bloxall, illustrating the "Plurality of Ringworm Fungi." Dr. Fox showed the three main forms which were found in London—the microsporon, the endothryx, and the ectothryx, and also some examples of favus-forming fungi—and demonstrated the difference in their appearances and modes of growth.

Dr. Bloxall described the culture experiments which they had made, showing the very different manner in which the different fungi developed in their non-parasitic phase, and exhibited some good photographs of their mode of inflorescence or spore formation while in this condition.

